# Effect of Chemical Degradation of Sodium Alginate on Capsaicin Encapsulation

**DOI:** 10.3390/molecules28237844

**Published:** 2023-11-29

**Authors:** Dominika Kulig, Łukasz Bobak, Andrzej Jarmoluk, Aleksandra Szmaja, Żaneta Król-Kilińska, Anna Zimoch-Korzycka

**Affiliations:** Department of Functional Food Products Development, Faculty of Biotechnology and Food Science, Wroclaw University of Environmental and Life Sciences, Chelmonskiego 37, 51-630 Wroclaw, Poland; dominika.kulig@upwr.edu.pl (D.K.); lukasz.bobak@upwr.edu.pl (Ł.B.); andrzej.jarmoluk@upwr.edu.pl (A.J.); aleksandra.szmaja@upwr.edu.pl (A.S.); zaneta.krol@upwr.edu.pl (Ż.K.-K.)

**Keywords:** sodium alginate, depolymerization, oligosaccharides, encapsulation, capsaicin

## Abstract

Capsaicin is known as an oily extract of paprika that is characterized by pungent taste and bioactivity. It also may cause irritation to the mouth and stomach which is why is so important to immobilize capsaicin on a carrier to prevent it. The usage of alginate oligomers, which has an antioxidant potential compared to alginate, is of benefit because it may be used in the immobilization of bioactive substances that are fragile to oxidation. The purpose of this study was to use sodium alginate oligomers as a coating material in the encapsulation process of paprika oleoresin. Alginate oligomers were produced by chemical degradation with hydrogen peroxide. The characteristics of the samples were obtained by measuring the viscosity, the contact angle of the surface, and the surface tension of solutions. The obtained solution of alginate oligomers served as the carrier material for the immobilization of capsaicin. Capsules were prepared by ionic gelation using a calcium chloride solution as a crosslinking agent. In this way, capsules without and with the core (capsaicin) were prepared and their ability to scavenge free radicals (DPPH) and iron-reducing properties (FRAP) were determined. The stability of the capsules was examined by thermal decomposition and under conditions of the gastric and small intestine, and capsaicin content was detected using high-performance liquid chromatography. It was found that alginate oligomers could be used in the encapsulation of bioactive compounds and the efficiency was above 80%. Capsule production from alginate oligomers affected their thermal stability. The use of alginate derivatives as a carrier increased the antioxidant properties of the finished product, as well as its ability to reduce iron ions. The use of alginate oligomers as a coating material prevented the active substance from being released too early in the conditions of the small intestine, prolonged the stability of the capsules, and supported their durability in gastric conditions.

## 1. Introduction

Due to its structure, alginate has the ability to gel, which is directly related to the number of G blocks in the chain. This is due to its ability to bind divalent metal cations. The calcium atom binds to the carboxyl groups of guluronic acid through ionic crosslinking. This gelation mechanism is called the “egg box” model [1]. The viscosity of the alginate increases as the pH of the solution decreases and reaches a maximum value in the range of pH 3.0–3.5. The molecular weight of alginates used on an industrial scale oscillates between 32,000 and 400,000 g/mol. Modifications that reduce the molecular weight improve the physical properties of the obtained alginate gels. On the other hand, an alginate solution obtained from a high molecular weight polymer has a high viscosity, which is a problematic parameter during further processing [2,3]. Therefore, sodium alginate can be degraded by various methods including irradiation (microwave, UV, X-ray, and gamma radiation), ultrasound, mechanical or thermal decomposition, oxidative degradation, and enzymatic or chemical hydrolysis. This treatment is most often performed to change the physical properties of alginate, primarily the decrease in molecular weight, which significantly improves its functional properties. Changing the spatial arrangement of alginate affects its mechanical strength, physiological and rheological properties, stability, viscosity, solubility in solutions, and hydrophobicity, and can also lead to an improvement in the reactive properties of the compound [4,5]. The sodium alginate oligosaccharides formed in the process of depolymerization do not have or have a very weak ability to form gels but are characterized by other important biological activities. They affect the growth and rapid development of human cells, including epidermal cells, and prevent oxidative stress. They favor the growth of plants such as rice, lettuce, wheat, and tobacco. Sodium alginate oligomers have antimicrobial properties and have a destructive effect on multidrug-resistant bacteria, which is why they are used in the production of antibiotics. They contribute to *Aspergillus* and *Candida* fungi [6]. Based on our ponder study, it was recognized that the degradation with H_2_O_2_ is the most effective chemical method for the obtained bioactive alginate oligosaccharides [7].

There are chemical, physical, and physicochemical methods for the encapsulation of active substances. Chemical methods include polycondensation, polymerization, and interfacial condensation. Physical coating mechanisms include spray drying, spheronization, and centrifugal extrusion, while physicochemical mechanisms include ionic gelation, coacervation, and extrusion [8]. The most popular way to form capsules is through the process of ionic gelation. This mechanism is based on interactions between polymers with different charges or the interaction of the polymer with a polycationic or polyanionic compound. Ion gelation consists of dropping the capsule solution into a water bath, thanks to which, through ionic interaction, the sol turns into a gel. When creating capsules, materials of natural origin including natural polymers such as agar, carrageenan, alginate, or chitosan are increasingly used [9]. It is recommended that the coating solution has the appropriate viscosity and concentration. By analyzing the available scientific articles, the coating material is most often present in a concentration of 0.5% to 2%. Because of its nontoxicity, biodegradability, and biocompatibility, alginate has become one of the main sources of coatings in the ion gel method. The advantage of using alginate for immobilization processes is its ability to form a gel in interaction with divalent metal ions, for example, Ca^2+^. The whole process takes place in a gentle and safe way, using room temperature. The simplest way to produce capsules is to dissolve or disperse the core substance in a solution containing the shell material, e.g., capsaicin in alginate dropped into the cross-linking aqueous medium. In the case of alginate, the preferred water bath is a solution of calcium chloride, in which the solution solidifies and capsules are formed. Another way is to form core-shell microcapsules. The process involves a separate dosing of the matrix and core material through the nozzle. The active substance is coated with the polymer solution immediately before contact with the cross-linking solution. As a result of this treatment, a capsule is obtained containing a separate part of the core (usually liquid) and the shell [10,11]. The concentration of calcium chloride in which the transition of alginate to gel form is observed is 0.1 M. The higher the concentration of ions in the crosslinking bath, the more Ca^2+^ ions pass into the capsule material during the mixing process. The final properties of the capsules are affected by the type of cross-linking agent, its concentration, the pH of the capsule formation reaction, and the temperature [9].

Capsaicin, the main bioactive substance of paprika oleoresin exhibits antioxidant activity but also has an inhibitory effect on bacterial growth. Extracts from plants of the genus Capsicum inhibit the development of microorganisms such as *Bacillus cereus*, *Bacillus subtilis*, *Clostridium sporogenes*, *Clostridium tetani*, and *Streptococcus pyogenes*. Its destructive effect has also been proven on some yeast species, e.g., *Saccharomyces cerevisiae*. The range of action of the alkaloid on microorganisms depends mainly on the concentration of the extract treated in a given colony and the resistance of the strain [12]. The antioxidant properties of capsaicin are used in the food industry to prevent reactions of fat oxidation, thus slowing down the degree of degradation of food products. Its influence on thermoregulatory processes, analgesic, anti-inflammatory, and anti-carcinogenic properties, and supporting the process of fighting obesity is used in medicine [12]. In the era of increasing consumer awareness and the search for alternative sources of food additives, capsaicin can be used to enrich food products or maintain food quality. The proposed capsaicin encapsulation system may also help to prevent irritation of the digestive tract and limit pungent taste.

The aim of this study was to use sodium alginate oligomers as a carrier in the encapsulation process of paprika oleoresin as the source of the bioactive substance, which is capsaicin. The encapsulation by alginate oligomers has not been reported so far. To characterize alginate oligomers, viscosity, contact angle, and surface tension tests were performed. Capsules from alginate oligomers were tested for encapsulation efficiency with high-performance liquid chromatography (HPLC), antioxidant effects, thermal treatment, and stability in gastric conditions and small intestines, confirmed by the HPLC method.

## 2. Results and Discussion

### 2.1. Solutions Characteristics

#### 2.1.1. Viscosity

The effect of hydrolysis time on the viscosity of alginate solutions was statistically significant and is shown in Table 1. As the hydrolysis time increased, the viscosity decreased significantly. The sodium alginate solution had the highest viscosity before the hydrolysis process began, which was 169.59 mPas (time 0 h). As degradation progressed, the viscosity of the solution decreased, with the highest rate of alginate degradation occurring in the first two hours of depolymerization, during which time the viscosity of the original solution decreased to 7.98 mPas. In the following hours of degradation (3 and 4 h), a statistically insignificant decrease in viscosity was noted compared to the first 2 h. The results recorded at these hydrolysis times were 7.14 mPas and 7.06 mPas, respectively. A significant decrease in viscosity value was noted after 24 h of degradation by H*_2_*O*_2_*. An empirical relationship between the logarithm of surface tension and the viscosity termed the fluidity was proposed by Pelofsky [13]. That is why the Pearson correlation between viscosity and surface tension of hydrolyzed sodium alginate was performed. The correlation was statistically significant and was 0.8346.

#### 2.1.2. Contact Angle

**Figure 1:**
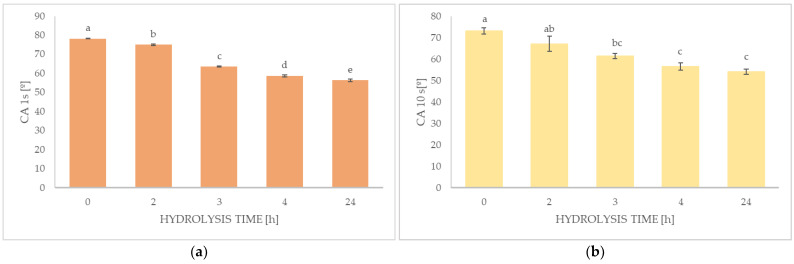
Effect of hydrolysis time: 0, 2, 3, 4, and 24 h on the contact angle of alginate sodium (A) solutions after 1 s (**a**) orange and 10 s (**b**) yellow from the drop deposition. ^a–e^ values with different letters within the same column differ significantly (*p* < 0.05). Results are expressed as the mean ± standard error.

Figure 1a,b presents the results of the statistical analysis of the influence of hydrolysis time on the value of the liquid contact angle (CA). It was observed that the hydrolysis time had a significant impact on the wetting properties of the surfaces of all the analyzed samples. As hydrolysis progressed, the value of the contact angle decreased (Figure 1a,b). The highest angle value measured after 1 s is for the 1% sodium alginate solution (78.230°), which means that it had the ability to wet the surface, but to the smallest extent compared to the other samples. The alginate solution had the best wetting properties on the Teflon surface after 24 h of oxidative degradation. The value of the measured parameter after hydrolysis was 56.397°.

The value of the contact angle is a measure of the wettability of the surface. The contact angle is defined as the angle between the plane of the solid surface and the tangent to the liquid surface drawn at the point of contact of the three phases. If the liquid has the ability to completely wet the surface of a solid, this angle takes the value 0. If the angle is less than 90°, the liquid has the ability to wet the surface, but it does not wet the surface in the range of 90°–180°. The hydrolysis process increased the surface wetting property of the sodium alginate solution. After repeating the measurement after 10 s, a decrease in the contact angle value was observed. Statistical analysis showed that the contact angle values measured after 10 s were no longer significant within the hydrolysis times of 0–2 h, 2–3 h, and 3–24 h. Because of the chemical degradation reaction, the polymer chains were shortened, which resulted in a decrease in the viscosity of the solution and, consequently, a reduction in the contact angle. Surface wettability has no effect on the duration of inertial wetting, but the viscosity of the liquid does [14]. Therefore, an increase in the liquid viscosity will increase the inertial wetting time, which confirms our observations.

#### 2.1.3. Surface Tension

The effect of hydrolysis time on surface tension (SFT) using the pendant drop method is shown in Table 2. The highest surface tension was found for a 1% sodium alginate solution at 78.297 mN/m. As hydrolysis progressed, the surface tension value decreased and in its final phase, it amounted to 69.133 mN/m. The hydrolysis time significantly affected the surface tension of the pendant drop. The essence of the method for determining the surface tension of liquids was the relationship between the shape of the liquid drop formed at the tip of the needle and its weight, as well as the corresponding equilibrium surface tension forces of the liquid. The acting surface tension forces led to the droplet becoming spherical, but under the influence of gravity, the liquid droplet became elongated. The surface tension depended on the molecular weight and viscosity. The higher the molecular weight, the higher the surface tension [15].

### 2.2. Capsules Characteristics

#### 2.2.1. Encapsulation Efficiency

The effect of the material used on the efficiency of capsaicin encapsulation was not statistically significant and the results for alginate sodium capsules with capsaicin was 89.33 ± 3.17%, and for capsules of alginate oligomers with capsaicin was 87.97 ± 2.23%. Belščak et al. observed that alginate capsules containing different plant extracts obtained using the same method also differed in process efficiency. The polyphenol extract of olive leaves had the highest efficiency (89.39%), while the nettle extract had the lowest efficiency (80.88%) [16]. The microencapsulation efficiency of fish oil with alginate was found to be greater than 80% [17]. The efficiency of the encapsulation process using the ion gelation method varies greatly, as the efficiency of the process depends on many factors, such as the concentration and type of alginate, the nature, and type of the immobilized substance, the concentration and type of cross-linking agents [18].

#### 2.2.2. Thermo-Gravimetric Analysis (TGA)

**Figure 2:**
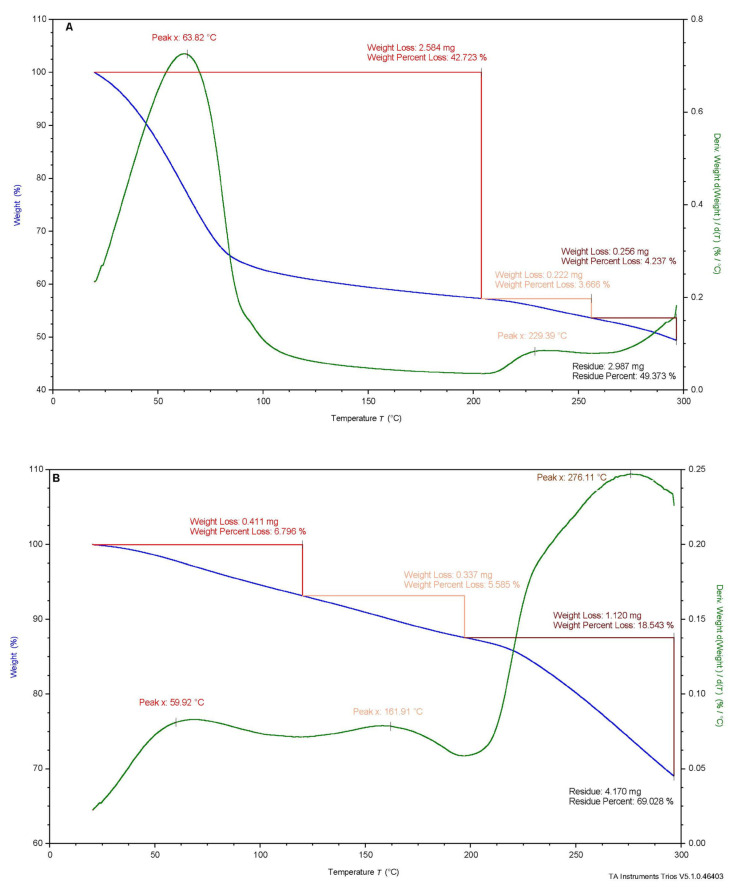

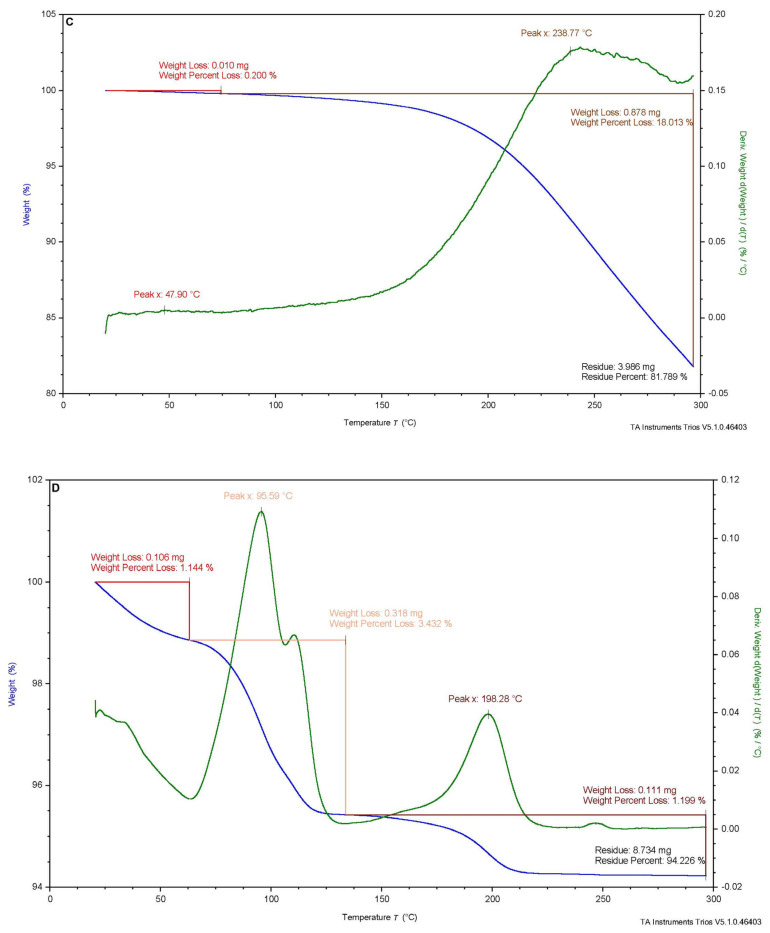
TGA decomposition curves of (**A**) AC (**B**) AOC (**C**) paprika oleoresin with 3.3% pure capsaicin (**C**,**D**) calcium chloride (CC).

The thermal decomposition of the AC (sodium alginate-capsaicin), AOC (sodium alginate oligomers-capsaicin) capsules, the paprika oleoresin with capsaicin (C), and the calcium chloride (CC) are presented in Figure 2. Thermal degradation of AC, AOC, and CC was visible as a three-stage process with residues of 49.373%, 69.028%, and 94.226%, respectively, while the decomposition of capsaicin was in one stage with a residue of 81.789%. It was observed that the TGA of alginate capsules with oleoresin had a great loss at 20–200 °C, corresponding to moisture evaporation. The next weight loss between 200 and 255 °C was due to the complexity of the process considered with sample degradation. The third weight loss was observed above 255 °C. Sodium alginate was decomposed by dehydration and degradation to sodium carbonate. Then at 550–750 °C, the material was carbonized and decomposed in nitrogen, which was observed by other authors [19,20,21]. The greatest weight loss of AOC was found in the third stage of thermal degradation between 220 and 300 °C. The percentages of residues of AC and AOC were about 49% and 69% respectively. When comparing total weight loss during thermal treatment, the AOC was more stable in a wide temperature range than AC. A great weight loss was found between 20 and 130 °C for the calcium chloride and corresponded to endothermic water removal. The second reduction was observed between 150 and 220 °C, which continued the endothermic dehydration of the material. When analyzing paprika oleoresin, thermal decomposition started around 150 °C and was related to volatile compounds. These results are in agreement with Pereda, Poncelet, and Renard [19]. 

#### 2.2.3. Antioxidant Properties

**Figure 3:**
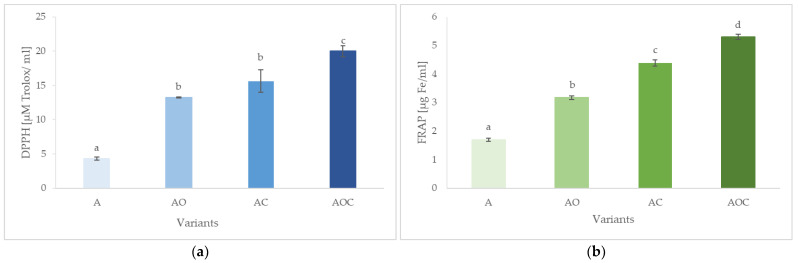
Antioxidant potential of (**a**) DPPH and (**b**) FRAP of AO—alginate oligomers capsules, A—alginate capsules, AC—alginate with capsaicin capsules, AOC—alginate oligomers with capsaicin capsules. ^a–d^ values with different letters within the same column differ significantly (*p* < 0.05). Results are expressed as the mean ± standard error.

The results of the statistical analysis illustrating the impact of the materials used for encapsulation on their antioxidant capacity as free radical scavenging activity and ferric reducing antioxidant power are presented in Figure 3a and Figure 3b, respectively. The type of core and shell substances used in the encapsulation processes affected the antioxidant properties of the final product. The lowest ability to scavenge DPPH free radicals had a sodium alginate solution in an amount of 4.319 µM of Trolox/mL. Statistical analysis did not show significant differences in the ability to remove free radicals for capsules with sodium alginate oligomers and double-core capsules with alginate and capsaicin. The degradation process allowed the improvement of the antioxidant properties of the analyzed capsule solutions. As a result, the alginate oligomer solution had the ability to neutralize free radicals at the level of 13.229 µM of Trolox/mL. The process of creating double core capsules using capsaicin as the core material and sodium alginate as the shell material allowed an increase in antioxidant activity to 15.618 µM of Trolox/mL. The highest ability to scavenge free radicals at the level of 19.979 µM of Trolox/mL, had capsules with sodium alginate oligomers in the capsaicin coating process. Capsaicin, thanks to its antioxidant properties, increases the ability to scavenge free radicals in capsules with sodium alginate and its oligomers. Amna et al. examined the antioxidant properties of a 3% capsaicin solution and polyurethane capsules with capsaicin. The determination of DPPH showed that a 3% capsaicin solution had a free radical scavenging capacity of 40%, while encapsulated capsaicin had a capacity of 42% [22]. 

The ferric reduction capacity was significantly variable and depended on the material and core used for the production of capsules (Figure 3b). The use of appropriate encapsulation materials affects the final ability of the capsules to reduce iron ions. The lowest FRAP value was obtained for the sodium alginate solution and was 1.695 µg of Fe/mL. After hydrolysis of sodium alginate using hydrogen peroxide, an increase in the ability to reduce ferric ions was observed, amounting to 3.166 µg of Fe/mL. Coated capsaicin with sodium alginate increased the FRAP value to 4.382 µg of Fe/mL compared to previous variants. However, the highest ferric-reducing antioxidant power (5.294 µg of Fe/mL) had AOC capsules. Furthermore, in our previous study, hydrolysis was observed to significantly improve the ferric-reducing antioxidant power, comparing native sodium alginate and sodium alginate oligomers [7].

#### 2.2.4. Stability of Capsules and Capsaicin Content under Gastric Conditions

No statistically significant changes were observed in the capsules during the process of exposing them to a solution that simulates gastric conditions. The capsules did not dissolve after 4 days of incubation in SGF solution at 37 °C. Additionally, capsaicin in AC and AOC capsules solutions was not detected (capsaicin content LOD < 0.10 µg/g) by HPLC analysis after 0, 24, 48, 72, and 96 h of incubation. The type of encapsulation material did not affect the stability of the capsules under gastric conditions. Gioumouxouzis et al. also found no visible changes in the appearance of alginate capsules after incubation in an acidic SGF solution. The carboxyl groups of alginate in the chain remain protonated, creating a tight polymeric network that hinders water exchange and circulation [23]. Therefore, the insolubility of alginate, alginate oligomers, and their variation with capsaicin capsules in SGF is related to the formation of an acid gel, which is enhanced by increasing the concentration of H+ [24].

#### 2.2.5. Stability of Capsules and Capsaicin Content under Conditions of the Small Intestine

While maintaining the analyzed capsule variants in the PBS solution, statistically significant changes in the dissolution rate were observed and are presented in Table 3. After 95 min, the alginate capsules without core dissolved in the PBS solution. Capsules made of sodium alginate oligomers obtained in the oxidative degradation process, as the shell material, were destabilized in the PBS solution after 1080 min, i.e., 18 h. It has been shown that the type of encapsulation materials used affects the dissolution time of the capsules under conditions of the small intestine. The use of sodium alginate oligomers as a capsule material significantly increased the time needed for their decomposition in the PBS environment. Visual observations were confirmed by HPLC analysis. The capsaicin content was not detected (capsaicin content below LOD < 0.10 µg/g) after 0, 30, 60 min in the AC capsules solution, and after 0, 30, 60, 90, 120, 960, 1020, 1080, 1140 min in the AOC capsules solution. The capsaicin was detected in the AC and AOC capsules solutions after 90 and 1080 min respectively and was between 0.10 and 0.32 µg/g. The assay examined the stability of capsules in the conditions of the small intestine and was carried out to check release of the active substance from the capsules. Gioumouxouzis et al. controlled the stability of the capsules in the conditions of the small intestine. After incubation in simulated intestinal fluid (SIF), it was observed that the alginate capsules dissolved after 90 min, while materials coated with sodium alginate destabilized after 150 min [23].

## 3. Materials and Methods

### 3.1. Materials

Sodium alginate with an M: G ratio of 1.4 and extracted from *Laminaria digitata* was supplied by Danisco, Grindsted, Denmark (particle size max. 5% > 400 µm). Paprika oleoresin with 3.3% pure capsaicin (C) was purchased from Essence, Konstancin-Jeziorna, Poland. It is a solution obtained by extracting the fruit of red chili paprika including oleoresin with derivatives of fatty acids and refined sunflower oil. Hydrogen peroxide (30%) was obtained from Pol-Aura, Morąg, Poland. Calcium chloride (CC) and ethanol (96%) were supplied by P.P.H. ‘STANLAB’ Sp. J., Lublin, Poland. Tween^®^80 and standard capsaicin were obtained from Sigma Aldrich, Poznań, Poland.

### 3.2. Sample Preparation

#### 3.2.1. Preparation of Alginate Oligosaccharides by Oxidation with Hydrogen Peroxide (H_2_O_2_)

The oxidation of sodium alginate was carried out using the method described in Zimoch-Korzycka et al. [7]. Sodium alginate (2% *w*/*v*) was dissolved in distilled water for 24 h using the R 50 CAT mechanical stirrer (Ballrechten, Dottingen, Germany) at 400 RPM. The degradation process was carried out using 10% hydrogen peroxide in an equal volume to 2% alginate sodium. The mixture was stirred at 600 RPM using a magnetic stirrer ECM CAT (Ballrechten-Dottingen, Germany) for 210 min at 25 °C. The solution obtained was freeze-dried with FreeZone 18L (Labconco, Kansas City, MO, USA) and stored as a lyophilized powder.

#### 3.2.2. Preparation of an Oleoresin Solution 

An emulsion of paprika oleoresin with a capsaicin content of 0.245% was created. For this purpose, a solution of deionized water (46.258 g) and 96% ethyl alcohol (46.258 g) was mixed in a 1:1 ratio, and 6.860 g of oleoresin and 0.624 g of Tween 80 were added. The solution was homogenized for 3 min at 3000 rpm using an IKA^®^ T18 ULTRA-TURRAX^®^ homogenizer (Staufen, Germany). The sample was left for 24 h to check the stability of the emulsion. 

#### 3.2.3. Encapsulation of Capsaicin in a Solution of Sodium Alginate Oligomers

The encapsulation process was carried out using the ‘BÜCHI’ B-390 encapsulator (Flawil, Switzerland). In this study, single-core capsules were produced using a 1.5% solution of sodium alginate oligomers and a 1.5% solution of sodium alginate. Furthermore, dual-core capsules of core-shell type in the capsaicin-alginate and capsaicin-sodium alginate oligomers were obtained. The carrier substance of the capsules was solutions with a concentration of 1.5%. For the encapsulation process, a head with a diameter of 300 μm was used for single-core capsules and a shell material for dual-core capsules. A 150 μm head was used to dispense capsaicin. The gelation process was carried out in a cross-linking bath using a 0.5 M solution of calcium chloride (CC) for 30 min. The capsules were washed with CC twice and then separated from the cross-linking agent using a ‘BÜCHI’ V-700 vacuum pump (Flawil, Switzerland). Table 4 shows the experimental design.

### 3.3. Methods

#### 3.3.1. Viscosity

The RheoStress 6000 rotary viscometer (Thermo Scientific Haake, Karlsruhe, Germany) was used for the rheological analysis. Viscosity was measured for a 1% sodium alginate solution, and 1% sodium alginate oligomers after 2, 3, 4, and 24 h of hydrolysis. Measurement was performed at 25 °C, using a cone sensor (C60/1_ Ti L, diameter 20) and measuring plate (TMP60 Steel 18/8) with an increasing shear rate of 1–1000 s^−1^ in linear distribution CR mode. Viscosity was determined at a shear rate of 100 s^−1^. Measurements were carried out in three repetitions for each sample.

#### 3.3.2. Contact Angle Measurement

The measurement of the contact angle was carried out using the sessile drop method using the DSA25 drop shape analyzer (KRÜSS, DSA 100 Hamburg, Germany). The tests were made for a 1% A solution (0 h of hydrolysis), and a 1% AO solution taken after 2, 3, 4, and 24 h of depolymerization. The drops were deposited on the Teflon material while maintaining a constant temperature of 20 °C. A 3.3 LS borosilicate glass 3.3 LS by MICROSYRINGES and an NE44 27749 needle with a diameter of 0.5 mm were used to dose the drops on the table surface. The determination was made in three repetitions for each tested sample.

#### 3.3.3. Surface Tension Using the Pendant Drop Method

The surface tension of a 1% A solution (0 h of hydrolysis) and AO after hydrolysis times of 2, 3, 4, and 24 h was determined using the pendant drop method. The drop shape analyzer DSA25 (KRÜSS, DSA 100 Hamburg, Germany) was used for the determination. The drop was dosed using a 3.3 LS MICROSYRINGES borosilicate glass syringe and an NE44 27749 needle with a diameter of 0.5 mm. The surface tension was measured in three repetitions for each sample.

#### 3.3.4. Determination of Encapsulation Efficiency

The encapsulation efficiency (EE) was determined according to the procedure of Hudita et al. and Kaiser et al. [25,26]. The amount of capsaicin loaded and filtrated was determined using the high-performance liquid chromatography method. A 10% solution of the tested capsules was prepared in a 10% sodium citrate solution to dissolve the capsules and release the immobilized capsaicin. The samples were sonicated (Hielscher UP100H sonicator, Teltow, Germany) to thoroughly dissolve the capsules. Then, extraction with hexane was performed 6 times. After hexane in reduced pressure, the residues were dissolved in 2 mL of methanol. The capsaicin content was determined in the samples prepared in this way. The analysis was performed using the HPLC technique using the Infinity 1220 II chromatograph from Agilent Technologies. The chromatographic analysis conditions were an isocratic flow of methanol 1 mL/min, using an XDB C18 column 150 × 4.6 mm (5 μm grain), and detection at a wavelength of λ = 228.4 nm with a reference wave of 360 nm and a slit of 100. The data collection speed was 1.25 Hz. Sigma-Aldrich capsaicin was used to prepare the standard curve. The capsaicin content in the sample was determined by the standard curve equation. The limit of detection (LOD) was determined to be 0.10 µg/g for capsaicin. The limit of quantification (LOQ) was determined to be 0.32 µg/g.

The efficiency was calculated according to the following formula:

$$EE=\frac{A_{loaded}-A_{filtrated}}{A_{loaded}}\;\cdot 100\%$$

where *EE* is encapsulation efficiency [%], *A_loaded_* is the amount of encapsulated capsaicin, and *A_filtrated_* is the amount of capsaicin in the ultrafiltrate.

#### 3.3.5. Thermal Gravimetric Analysis (TGA)

The thermal stability of AC and AOC, C, and CC was measured by thermal gravimetric analysis using a TGA 5500 thermogravimetric analyzer from TA Instruments Company (Tokyo, Japan). The analysis temperature was performed from room temperature to 300 °C in an inert nitrogen atmosphere with a flow rate of 25 mL/min and a warming rate of 10 K/min according to Kulig et al. [27].

#### 3.3.6. Antioxidant Properties

##### Free Radical Scavenging Activity (DPPH)

The free radical scavenging activity of DPPH was determined spectrophotometrically (UviLine 9400 SI Analytics, Mainz, Germany) at a wavelength of 517 nm according to Chen et al. [28]. The study was carried out for A and AO after 24 h of hydrolysis and a homogenized solution of alginate-capsaicin and oligomer-capsaicin capsules. A total of 1 mL of a 0.1% solution of the test sample and 1 mL of 96% ethyl alcohol were placed in the test tubes. The solutions were mixed using a Vortex V-1 plus and then 0.5 mL of a 0.3 mM ethanolic DPPH radical solution was added to the samples. The samples were incubated for 30 min. The assay was performed in triplicate. The reagent sample was a mixture of 1 mL of H_2_O, 1 mL of ethanol, and 0.5 mL of a 0.3 mM ethanol solution of DPPH radicals. The results were calculated based on the standard curve expressed in units of μg of Trolox needed to neutralize a 0.3 mM solution of DPPH radicals.

##### Ferric Reducing Antioxidant Power (FRAP) 

The determination was carried out according to Benzie and Strain [29]. The study was carried out for a 0.1% sodium alginate solution, 0.1% sodium alginate oligomers, and a homogenized 0.1% solution of alginate-capsaicin and oligomer-capsaicin dual core capsules. For this purpose, 3 mL of the reagent was added to 1 mL of the test sample and the samples were incubated for 10 min. The absorbance was performed on a UviLine 9400 (SI Analytics, Mainz, Germany) spectrophotometer using a wavelength of 593 nm. The reagent test was a mixture of 1 mL of deionized water with 3 mL of working reagent. The assay was carried out in three repetitions. The content of Fe^2+^ ions in the sample was calculated on the graph of the standard curve graph.

#### 3.3.7. Stability of Capsules under Gastric Conditions

The resistance of the capsules to the conditions of the stomach was tested using the SGF (Simulated Gastric Fluid, St Charles, MO, USA) solution. The solution was prepared by adding 2 g of sodium chloride and 7 g of hydrochloric acid to 1000 mL of deionized water. By treatment with 0.1 M hydrochloric acid, the solution was adjusted to a pH of 1.2 [30,31]. The determination was carried out for single-core capsules made of sodium alginate and sodium alginate oligomers and, for dual-core capsules, sodium alginate–capsaicin, and oligomers–capsaicin. For this purpose, 1 g of capsules was introduced into 20 mL of SGF solution. The samples were placed in a water bath with a shaking function, with a temperature of 37 °C and a speed of 95 rpm. The degree of dissolution of the capsules was visually controlled for 4 days. Analysis was performed in triplicate for each of the samples. Additionally, the capsaicin content was monitored based on the high-performance liquid chromatography method described in 3.3.4 after 0, 24, 48, 72, and 96 h. 

#### 3.3.8. Stability of Capsules in Small Intestine

The resistance of the capsules to the conditions of the small intestine was tested using a PBS (Phosphate Buffered Saline) solution. The solution was prepared by adding 8 g of sodium chloride, 0.2 g of potassium chloride, 1.44 g of sodium hydrogen phosphate, and 0.24 g of potassium dihydrogen phosphate to 800 mL of deionized water. By dosing a 0.1 M HCl solution, the pH of the solution was adjusted to 7.4. The solution was supplemented with water to a volume of 1000 mL [32]. The determination was carried out for single-core capsules made of sodium alginate and sodium alginate oligomers and, for dual-core capsules, sodium alginate–capsaicin, and oligomers–capsaicin. For this purpose, 1 g of capsules was introduced into 20 mL of PBS solution. The samples were placed in a water bath with a shaking function, with a temperature of 37 °C and a speed of 95 rpm. The stability of the capsules was visually checked. Analysis was performed in triplicate for each of the samples. Additionally, the HPLC method described in 3.3.4 was used to detect capsaicin after 0, 30, 60, 90, 120, 960, 1020, 1080, and 1140 min. 

#### 3.3.9. Statistical Analysis

The results obtained were statistically analyzed in the Statistica 13.3 program (StatSoft, Krakow, Poland). For this purpose, a one-way analysis of variance was performed using the Duncan test at the significance level α ≤ 0.05. Additionally, a Pearson correlation was used to analyze the results of viscosity and surface tension at the significance level α ≤ 0.05. 

## 4. Conclusions

The chemical degradation process affects the physical and chemical properties of the sodium alginate solution. Sodium alginate oligomers formed during the chemical degradation can be used as shell material in capsaicin encapsulation processes with similar efficiency. Double-core capsules made from a solution of alginate oligomers and capsaicin had the highest ability to scavenge DPPH free radicals. The use of a solution of alginate oligomers as a coating material allowed for an increase in the ferric-reducing antioxidant power. The capsules obtained, regardless of the encapsulation materials, were stable in gastric conditions. The use of sodium alginate oligomers increased the resistance of capsules to the conditions of the small intestine, expanding the possibilities of their use for the controlled release of drugs or bioactive substances. The thermal stability of the formed capsules allows them to be used in food production where thermal processes are required.

## Figures and Tables

**Table 1:** Effect of hydrolysis time: 0, 2, 3, 4, and 24 h on the viscosity of alginate solutions.

Hydrolysis Time [h]	Viscosity [mPas]
0	249.590 ^a^ ± 2.115
2	17.985 ^b^ ± 0.412
3	17.141 ^b^ ± 0.253
4	17.065 ^b^ ± 0.191
24	16.327 ^c^ ± 0.291

^a–c^ values with different letters within the same column differ significantly (*p* < 0.05). Results are expressed as the mean ± standard error.

**Table 2:** Effect of hydrolysis time: 0, 2, 3, 4, and 24 h on the surface tension of alginate solutions.

Hydrolysis Time [h]	SFT [mN/m]
0	78.297 ^a^ ± 0.176
2	74.680 ^b^ ± 0.278
3	71.187 ^c^ ± 0.240
4	70.510 ^d^ ± 0.392
24	69.133 ^e^ ± 0.450

^a–e^ values with different letters within the same column differ significantly (*p* < 0.05). Results are expressed as the mean ± standard error.

**Table 3:** Effect of the type of capsule material and the core used (AO—alginate oligomers capsules, A—alginate capsules, AC—alginate with capsaicin capsules, AOC—alginate oligomers with capsaicin capsules) on stability and capsaicin content under the conditions of the small intestine.

Variants	Stability Time [min]	Capsaicin Content [µg/g]
AO	1080.00 ^b^ ± 2.00	-
A	95.00 ^a^ ± 1.00	-
AC	92.00 ^a^ ± 5.00	0.10–0.32 *
AOC	1075.00 ^b^ ± 5.00	0.10–0.32 **

^a,b^ values with different letters within the same column differ significantly (*p* < 0.05). Results are expressed as the mean ± standard error. * mean capsaicin content after 90 min of incubation. ** mean capsaicin content after 1080 min of incubation.

**Table 4:** Experimental design.

Coding	Alginate [A] Concentration [%]	Alginate Oligomers [AO] Concentration [%]	Capsaicin [C] Content [%]
AO	-	1.5	-
A	1.5	-	-
AC	1.5	-	0.245
AOC	-	1.5	0.245

## Data Availability

The data presented in this study are available on request from the corresponding author.
